# Tissue Expression Difference between mRNAs and lncRNAs

**DOI:** 10.3390/ijms19113416

**Published:** 2018-10-31

**Authors:** Lei Chen, Yu-Hang Zhang, Xiaoyong Pan, Min Liu, Shaopeng Wang, Tao Huang, Yu-Dong Cai

**Affiliations:** 1 School of Life Sciences, Shanghai University, Shanghai 200444, China; chen_lei1@163.com (L.C.); wsptfb@163.com (S.W.); 2 College of Information Engineering, Shanghai Maritime University, Shanghai 201306, China; liumin@shmtu.edu.cn; 3 Shanghai Key Laboratory of PMMP, East China Normal University, Shanghai 200241, China; 4 Institute of Health Sciences, Shanghai Institutes for Biological Sciences, Chinese Academy of Sciences, Shanghai 200031, China; zhangyh825@163.com; 5 Department of Medical Informatics, Erasmus MC, 3000 CA Rotterdam, The Netherlands; xypan172436@gmail.com

**Keywords:** mRNA, lncRNA, expression specificity, cell type, feature selection

## Abstract

Messenger RNA (mRNA) and long noncoding RNA (lncRNA) are two main subgroups of RNAs participating in transcription regulation. With the development of next generation sequencing, increasing lncRNAs are identified. Many hidden functions of lncRNAs are also revealed. However, the differences in lncRNAs and mRNAs are still unclear. For example, we need to determine whether lncRNAs have stronger tissue specificity than mRNAs and which tissues have more lncRNAs expressed. To investigate such tissue expression difference between mRNAs and lncRNAs, we encoded 9339 lncRNAs and 14,294 mRNAs with 71 expression features, including 69 maximum expression features for 69 types of cells, one feature for the maximum expression in all cells, and one expression specificity feature that was measured as Chao-Shen-corrected Shannon’s entropy. With advanced feature selection methods, such as maximum relevance minimum redundancy, incremental feature selection methods, and random forest algorithm, 13 features presented the dissimilarity of lncRNAs and mRNAs. The 11 cell subtype features indicated which cell types of the lncRNAs and mRNAs had the largest expression difference. Such cell subtypes may be the potential cell models for lncRNA identification and function investigation. The expression specificity feature suggested that the cell types to express mRNAs and lncRNAs were different. The maximum expression feature suggested that the maximum expression levels of mRNAs and lncRNAs were different. In addition, the rule learning algorithm, repeated incremental pruning to produce error reduction algorithm, was also employed to produce effective classification rules for classifying lncRNAs and mRNAs, which gave competitive results compared with random forest and could give a clearer picture of different expression patterns between lncRNAs and mRNAs. Results not only revealed the heterogeneous expression pattern of lncRNA and mRNA, but also gave rise to the development of a new tool to identify the potential biological functions of such RNA subgroups.

## 1. Introduction

Ribonucleic acid (RNA) is one of the most significant nucleic acid components in all living creatures except for some viruses [1,2]. In general, nucleic acid is one of the three major macromolecules that maintain the fundamental biological processes of all known forms of life [2]. Among the different subtypes of nucleic acid, RNA is a specific functional component that has specific structures and functions. As for the structures, generally, DNA, as another major component of nucleic acid in our living cells, exists as desoxy-ribonucleic acid of double-strand status with bases as A, G, C, and T in the cell nucleus as the transporter of genetic materials [3]. However, RNA that plays different biological roles generally acts as single-strand ribonucleic acid, or in other words, a chain of ribonucleic acid with specific base U but not T [4,5,6]. Aside from the structure differences, the biological functions of RNA are definitely specific [7]. Usually, RNA contributes to the maintenance and regulation of gene expression and function, regulating the coding, decoding, transcription, and translation of a specific protein, the final functional component of most biological processes; these factors are quite different from the biological functions of other nucleic acid subtypes, such as DNA, as the genetic passenger in the cell nucleus [7].

Similar to other material categories, RNA is also not a consistent group of subtype members with similar structure and functions. Messenger RNA (mRNA) and long noncoding RNA (lncRNA) are the two main subgroups of RNAs that participate in transcription regulation [8,9]. In general, RNA can be divided into two main subgroups, namely, coding RNA (mRNA) and noncoding RNA (ncRNA), according to their basic biological function, whether they can be further translated into proteins. For a long time, investigators mainly focus on the mRNA with specific protein-coding potentials and have been considered to be the only functional component of RNAs in living cells for a long time [9]. However, with the development of biotechnologies and the deepening of understanding on mRNA transcription, translation, and protein coding, ncRNAs are obtaining considerable attention in multiple biological research fields due to their irreplaceable regulatory roles of such biological processes [10,11,12]. Among these ncRNAs, lncRNAs turn out to be one of the particular subgroups with specific biological structures and functions.

In general, lncRNAs refer to nonprotein coding RNAs that are longer than 200 nucleotides [11,13]. Considering the distinctive structure of lncRNAs, which is different from other regulatory ncRNAs with quite short sequences similar to microRNAs (miRNAs) and short-interfering RNAs (siRNAs), lncRNAs have specific biological functions and, therefore, may be quite irreplaceable for the physical regulation [14,15,16]. Gene transcription regulation, post transcriptional regulation, and epigenetic regulation are the three main biological functions of lncRNAs according to existing literature [17,18,19,20]. The major biological functions of lncRNAs are gene-specific transcription regulation, which can be concluded into the gene transcription regulation subgroup, considering the specific gene *TP53*, a famous tumor suppressor, as an example. Recent publications confirmed that natural antisense transcripts of *TP53*, named *Wrap53*, which is also a functional lncRNA, directly participates in the regulation of *TP53* transcription and translation by targeting the 5′ untranslated region of *TP53*, enhancing the expression of such gene; this condition implies the gene-specific regulatory function of lncRNAs [21,22,23]. Considering that lncRNAs may have gene specific regulatory functions in living cells, as a regulator, lncRNAs may also have cell-type specific expression pattern, corresponding to their mRNA targets and reflecting biological functional features of such cell type similar to the cell-type specific expression of functional genes. Although lncRNAs have been widely reported to be multifunctional and play an irreplaceable role in various biological processes, identifying the distributive features and expression pattern of lncRNAs, especially the cell-type specific expression pattern, is still quite difficult.

In this study, we tried to identify specific cell types, in which lncRNAs may play the most irreplaceable roles. Considering that lncRNAs tend to have lower expression level comparing to coding RNAs, the direct comparison of lncRNA expression level with detailed fragments per kilobase million (FPKM) may not be suitable for further cell typing in this study, introducing systematic errors. Therefore, we introduced a specific benchmark for further screening. This method turns relies on the dissimilarity of the lncRNA and mRNA expression pattern. A recent study on the expression pattern of human lncRNAs confirmed the cell-type specific lncRNA expression pattern [24]. For such dataset, we proposed a computational scheme incorporating several advanced computational methods, such as maximum relevance minimum redundancy (mRMR) method [25], incremental feature selection (IFS) method [26], random forest (RF) algorithm [27], and repeated incremental pruning to produce error reduction (RIPPER) algorithm [28], to analyze this dataset. For the first time, we identified a group of cell types as well as expression pattern features and rules that may be associated with distinctive expression pattern of lncRNAs and mRNAs, contributing to the identification of lncRNA specific expression pattern and further revealing the potential biological functions of lncRNAs.

## 2. Results

**Figure 1:**
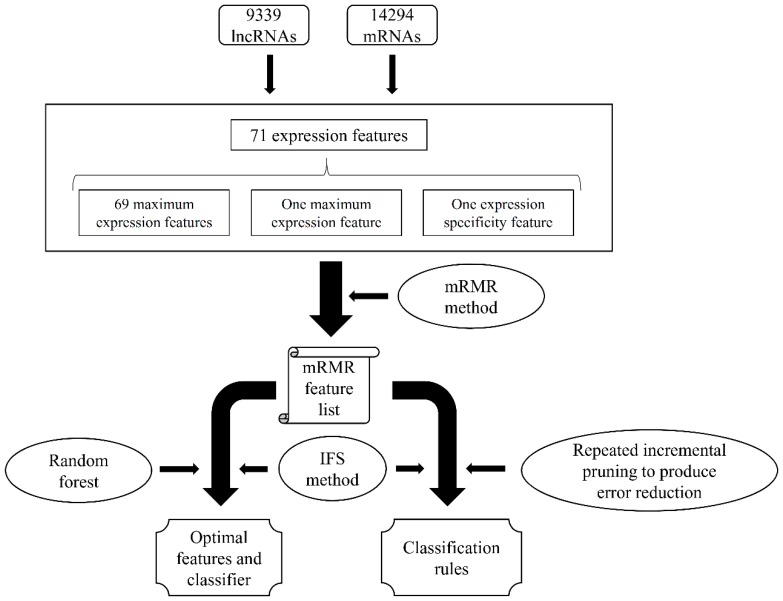
Entire procedures of the computational scheme for investigating lncRNA and mRNA with expression features. The 71 expression features were analyzed by the maximum relevance minimum redundancy (mRMR) method, resulting in an mRMR feature list. Then, the incremental feature selection (IFS) method with the random forest (RF) algorithm was used to extract the optimal features and build the optimal RF classifier. At the same time, the IFS method with the repeated incremental pruning to produce error reduction (RIPPER) algorithm was adopted to learn classification rules.

In this study, we built a computation scheme to analyze the expression features of lncRNAs and mRNAs, trying to extract essential differences between these two types of RNAs. The entire procedures are illustrated in Figure 1.

### 2.1. Results of the Maximum Relevance Minimum Redundancy (mRMR) Method

Each lncRNA or mRNA was encoded into 71 expression features that are described in detail in Section 4.1. Then, a popular feature selection procedure, mRMR method [25], was applied to analyze these features, producing the mRMR feature list, wherein the 71 features were sorted according to their relevance to target variable and redundancies to other features. The obtained mRMR feature list is presented in Table S1.

### 2.2. Results of the Incremental Feature Selection (IFS) Method with Random Forest (RF)

In the mRMR feature list, features were sorted in descending order according to their importance on discriminating lncRNAs from mRNAs. Based on this view, the combination of some top features in the list can yield accurate prediction. Thus, we applied the IFS method, together with RF as classification algorithm, to extract the optimal combination of features, thereby building an optimal RF classifier. In detail, we constructed 71 feature subsets according to the mRMR feature list, each of which contained some top features in the list. Then, for each feature subset, all RNAs were represented by features in this set and a RF classifier was built on them. A 10-fold cross-validation was adopted to evaluate the performance of each classifier. The prediction performances of classifiers represented by the four measurements (sensitivity (SN), specificity (SP), accuracy (ACC), and Matthew’s correlation coefficient (MCC)) are listed in Table S2.

**Figure 2:**
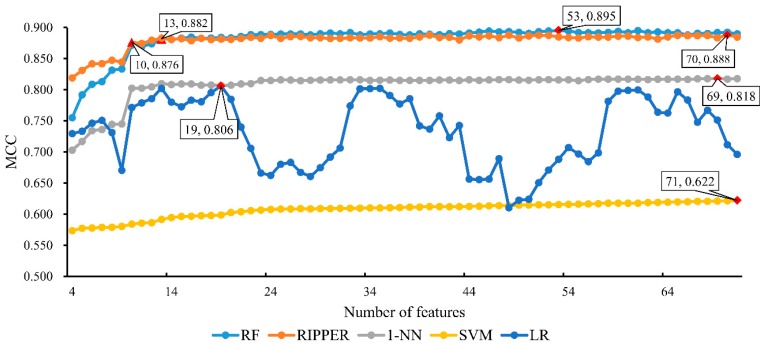
IFS curves based on the predicted results yielded by the IFS method with five different classification algorithms. The X-axis represents the number of features participating in the classification, and the Y-axis represents the Matthew’s correlation coefficients (*MCC*s). The optimal *MCC* (marked with red diamonds) for random forest (RF), repeated incremental pruning to produce error reduction (RIPPER), nearest neighbor algorithm (1-NN), support vector machine (SVM) and logistic regression (LR) is 0.895, 0.888, 0.818, 0.622 and 0.806, respectively. For RF, when top 13 features were used, the *MCC* value first overcomes 0.880. While for RIPPER, the MCC value first achieves 0.870 when top 10 features were employed.

For ease of observation, a curve, named IFS curve, was plotted by setting the *MCC* values as the Y-axis and the number of features participating in building classifiers as the X-axis. As shown in Figure 2, *MCC* reaches the optimal value of 0.895 when the first 53 features were used in classification. Accordingly, these 53 features were picked up as optimal features for RF. In addition, the corresponding RF classifier, using these features to represent lncRNAs and mRNAs, was denoted as the optimal RF classifier. Moreover, the optimal classifier yielded the SN of 0.963, SP of 0.940, and ACC of 0.949, listed in Table 1. This result indicated that the constructed optimal classifier performed well.

### 2.3. Results of the IFS Method with Repeated Incremental Pruning to Produce Error Reduction (RIPPER)

Based on the IFS method and RF, we built an optimal RF classifier with good performance (MCC = 0.895). However, this classifier was a black box, which cannot clearly indicate the differences between lncRNAs and mRNAs. In view of this, the rule learning algorithm, RIPPER algorithm, was adopted to do the same procedures that were done for RF. The purpose was to extract informative rules that can give a clear picture on different expression patterns between lncRNAs and mRNAs.

Similar to the IFS method with RF, for each of constructed feature subsets, we constructed an RIPPER classifier, which contained several classification rules, and evaluated its performance with 10-fold cross-validation. The performance of these classifiers is listed in Table S3. For easy observation, we also plotted an IFS curve in Figure 2. It can be observed that when the first 70 features were used to construct the RIPPER classifier, it yielded the best MCC of 0.888, which was slightly lower than that yielded by the optimal RF classifier. Accordingly, the optimal RIPPER classifier was built using the first 70 features in the mRMR feature list. The detailed performance, including SN, SP and ACC, is listed in Table 1. Compared with the performance of the optimal RF classifier, RIPPER classifier yielded higher SP and lower SN, ACC and MCC. However, they were only slightly lower than those of the optimal RF classifier. Thus, the performance of the optimal RIPPER classifier was competitive compared with optimal RF classifier.

### 2.4. Comparison of the IFS Method with Other Classification Algorithms

As mentioned above, we selected the RF as the classification algorithm for constructing the best classifier. From the results in Section 2.3, we can see that RF was slightly superior to RIPPER. Here, we further employed other three classification algorithms, namely, nearest neighbor algorithm (1-NN) [29], support vector machine (SVM) [30], and logistic regression (LR) [31], to clarify that RF is a proper choice. To this end, we employed three tools, called “IBk”, “SMO” and “Logistic”, in Weka, which implement the aforementioned algorithms. For convenience, these three tools were executed with their default parameters. The procedures for these three algorithms were almost the same as those for RF and RIPPER. The performance of these three algorithms on the constructed feature subsets is listed in Table S4. In addition, an IFS curve was plotted for the results obtained by each algorithm, as shown in Figure 2. The highest MCC for 1-NN was 0.818 when top 69 features in the mRMR feature list were used, whereas for SVM, the highest MCC was 0.622 when all features (71 features) were used, and the highest MCC was 0.806 for LR, which was obtained based on 19 features in the mRMR feature list. Obviously, MCCs yielded by these three algorithms were all much lower than that yielded by the optimal RF classifier. The detailed performance of the optimal classifiers based on five classification algorithms is listed in Table 1, from which we can see that all four measurements produced by the optimal RF classifier were higher than those obtained by the optimal 1-NN, SVM and LR classifier. This finding suggested that the selection of the RF as the classification algorithm is a good choice. Furthermore, Figure 2 shows that the performance of the RF classifier based on each constructed feature subset is better than that of other three classifiers because the IFS curve of RF is always above the IFS curves of 1-NN, SVM and LR. This outcome further proved the abovementioned conclusions.

## 3. Discussion

### 3.1. Analysis of Important Features for Constructing Optimal RF Classifier

**Figure 3:**
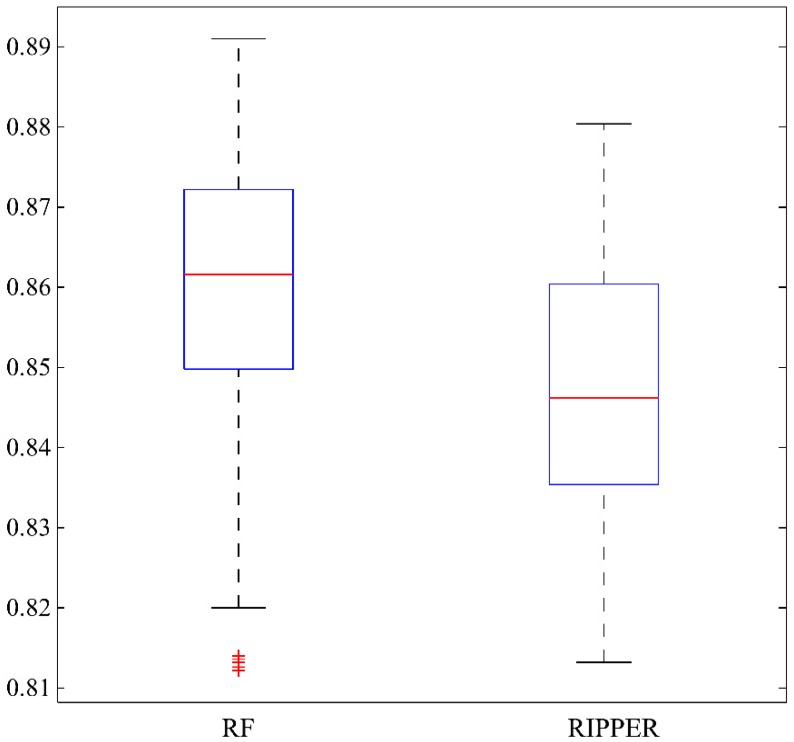
A box plot to illustrate the performance, measured by Matthew’s correlation coefficient, of random forest (RF) and repeated incremental pruning to produce error reduction (RIPPER) algorithms on 1000 randomly produced feature subsets. For RF, each feature subset contained the feature of expression specificity and 12 other features randomly selected from rest 70 features, while for RIPPER, each feature subset contained the feature of expression specificity and 9 other features randomly selected from rest 70 features.

As mentioned in Section 2.2, we extracted 53 optimal features for RF. Among them, the most were regarding cell types, wherein two types of RNAs might express differentially. However, analyzing all these features one by one and finding the central tendency of cell types are quite difficult. Meanwhile, these processes are helpful to discriminate different expression patterns of the two RNA types. By observing Figure 2, when top 13 features (listed in Table 2) were used to construct the RF classifier, the *MCC* value first overcomes 0.880 that was almost equal to that of the optimal RF classifier. Among these 13 features, one feature is expression specificity, describing the general gene-based expression levels and specificity in primary cell facets. To indicate the statistical significance of these 13 features, we randomly constructed 1000 feature subsets, each of which contained the feature of expression specificity and 12 other features randomly selected from rest 70 features. Based on each of these feature subsets, we built a RF classifier and evaluated its performance with 10-fold cross-validation. Accordingly, we accessed 1000 *MCC*s and draw their box plot in Figure 3, from which we can see that 0.880, yielded by the first 13 features in the mRMR feature list, lies at the top of the box plot. Furthermore, the mean and standard deviation of 1000 *MCC*s were 0.860 and 0.015. 0.880 was larger than 0.860 + 1.3 × 0.015, suggesting it was high with statistical significance. Therefore, these 13 features contained essential information about cell types that can be used to distinguish lncRNAs from mRNAs and are reviewed in this section. 

Among such top 13 features, 11 features are about detailed cell subtypes, in which lncRNAs and mRNAs may have quite different expression patterns. This condition implied that such cell subtype may be a potential benchmark for further identification of specific lncRNA functions. As for the two remaining features, one of them, namely, expression specificity, describes the general gene-based expression levels and specificity in primary cell facets. This aspect is definitely associated with lncRNA regulation. Another feature, named max cpm (count per million) in all facets, describes the detailed expression quantity of either lncRNAs or mRNAs. Therefore, such regulatory factor may also be quite essential for the distinction of lncRNA and mRNA, considering the distinctive expression pattern of such two RNA subtypes according to recent publications [32,33,34]. Based on recent publications, all top 13 features, or in other words, cell subtypes can be confirmed to have specific lncRNA and mRNA expression pattern. The detailed analysis can be seen below.

Among the top 13 features, 11 were involved in cell subtypes that were determined to have specific distinctive lncRNA expression pattern compared with respective mRNAs. This condition indicated that in such cell subtypes, lncRNA may play specific biological roles, which can be validated by recent publications. Among them, intestinal epithelial cell (with rank 2 in the mRMR feature list) was deemed to be the optimal cell type with distinctive lncRNA expression pattern. Recent publications confirmed that various lncRNAs are upregulated in the intestinal epithelial cells, contributing to various gene expression regulations in response to environmental stimulus [35,36]. In general, intestinal epithelial cell directly interacts with the exogenous diet. In response to the stimulus of exogenous food or microbes, some certain biological functions of intestinal epithelial cells need to be modulated in time [37]. However, considering that the pre-transcriptional regulation of gene expression is quite hard and time consuming, lncRNAs as a functional component of post-transcription regulation may definitely act as a major regulator against such stimulus, resulting in the distinctive and flexible expression pattern of lncRNAs and mRNAs [38,39]. Aside from intestinal epithelial cell, which is a functional component of the digestive system, another specific cell subtype (hepatocyte) may have its respective lncRNA expression pattern, as compared with the mRNA expression of itself. In 2014, a specific study on the expression pattern of lncRNAs in the liver tissue revealed that during the developmental processes, the expression pattern of lncRNAs may change accordingly, participating in various regulatory processes for liver maturation [40]. Similar to intestinal epithelial cells, hepatocytes also have distinctive lncRNA expression pattern. However, this pattern can attribute to two major inducements, namely, exogenous and endogenous. As for the endogenous factors, in such literature, during the liver development, lncRNA has to be expressed highly flexibly for the accurate expression regulation in time, resulting in the distinctive expression pattern [40]. Exogenous factors, on the contrary, affect the liver maturation. Recent publications also confirmed that as the major organs/cell subtypes for biological transformation, hepatocyte may also have different lncRNA expression pattern after the impaired transformative function induced by partial hepatectomy [41]. Therefore, similar to intestinal epithelial cells, hepatocyte may also have its specific lncRNA expression pattern due to the fickle exogenous stimulus. Similar mechanisms may also be applied to conjunctiva fibroblast, mesenchymal cells, and pericyte cells that mainly interact with the stimulus factors either internal or external [42,43,44,45].

Aside from such epithelial or mesenchymal cells that usually have specific lncRNA expression pattern compared with mRNA due to the exogenous or endogenous stimulus factors, five specific stimulus-responsive cell subtypes are available that can be clustered into another group due to their specific immune-associated functions. Different from the aforementioned cell subtypes that interact with stimulus factors, immune cells, including five cell subtypes we extracted, may actively identify and respond to the exogenous or endogenous stimulus usually as antigens. Neutrophil, as one of the major types of granulocytes in the white blood cell subgroups, has been widely reported to have specific reflective pattern against endogenous or exogenous stimulus [46,47,48]. During the regulatory processes, especially the modulation against exogenous stimulus like pathogens, lncRNA has also been reported to be differentially regulated, inducing a specific relative expression pattern of lncRNA compared with mRNAs in this cell type [49,50,51]. Actually, lncRNAs have been reported to be quite functional and regulatory in multiple immune-associated cells, such as macrophage, dendritic cells, neutrophils, and lymphocytes (both B and T cells), confirming the prediction of B cells and macrophage [52,53]. As for mast cells, another functional component of immune-associated cells, recent publications on the lncRNA expression pattern of such cell subtype directly confirmed that the expression of lncRNAs in mast cells may modulate the inflammatory and immune recognition characteristics of such cells [54]. During the inflammatory processes induced by either tumorigenesis or exogenous infections, the unique expression pattern of certain functional lncRNAs compared with mRNAs may contribute to the accurate and controllable immune response against exogenous or endogenous stimulus [54].

Two unique non-terminally differentiated cells, namely, reticulocytes and neuronal stem cells, relating to two optimal features exist. Reticulocytes, as the immature status of erythrocytes, have been widely reported to have a specific lncRNA expression pattern, contributing to the maturation and enucleation processes [55,56]. Considering that during the maturation of reticulocytes, the mRNA composition of such cell types changed from multicomponent to single component and the hemoglobin transcription [57]. As for the lncRNAs, recent publications also confirmed that during the maturation processes, lncRNAs maintained in the erythrocytes can consistently regulate their respective biological functions, preventing the mature red blood cells from death [58]. Therefore, the expression pattern of lncRNAs and mRNAs of reticulocytes may both be different from other cell types. As for the remaining cell subtype, namely, neuron stem cells, these cells are quite a heterogeneous population, including quiescent neural stem cell, active neural stem cell, niche astrocytes, and neural progenitor cells [59,60]. Recent publications also confirmed that the mRNA and lncRNA expression pattern of neural stem cells may be quite heterogeneous. This finding implied the unique RNA expression pattern of such two cell subtypes.

### 3.2. Analysis of Classification Rules Yielded by RIPPER

In Section 2.3, we built an optimal RIPPER classifier with top 70 features in the mRMR feature list. Based on these features, RIPPER can produce 31 rules for classifying lncRNAs and mRNAs. These rules were too many for us to analyze them one by one. In view of this, we carefully checked the IFS curve of RIPPER in Figure 2 and found that when the top ten features were selected, the corresponding RIPPER classifier can yield an MCC of 0.876, which was quite close to the MCC of the optimal RIPPER classifier. Similar to RF, we also tested the statistical significance of these ten features. 1000 feature subsets with 10 features were randomly produced, each of which contained the feature of expression specificity and nine features randomly selected from rest 70 features. The 1000 RIPPER classifiers were built on these feature subsets, which were further evaluated via 10-fold cross-validation. Based on the obtained 1000 MCCs, a box plot was drawn in Figure 3. It can be observed that 0.876, obtained by the first ten features in the mRMR feature list, also lies at the top of the box plot. In addition, we also counted the mean and standard deviation of these 1000 MCCs, obtaining the mean of 0.847 and the standard deviation of 0.015. 0.876 was higher than 0.847 + 1.8 × 0.015, implying the MCC yielded by the first 10 features in the list was high with statistical significance. Thus, we selected these ten features to produce classification rules via RIPPER, obtaining 18 rules listed in Table 3. This section gave some analyses on these rules. According to recent publications, several rules can be confirmed. Here, we screened five rules for detailed analysis and the remainder of the rules were left to readers.

The first rule (Rule-1) involves the RNA expression pattern of four tissues and the expression specificity described by Chao-Shen-corrected Shannon’s entropy, reflecting the randomized expression pattern of certain RNA subtypes. The higher such parameter that describing expression specificity is, the more exclusively such screened out RNA may be expressed and the higher expression specificity such RNA may have [61]. Lower expression of RNA in four tissues (neuronal stem cell, mesenchymal cell, intestinal epithelial cell and mesenchymal cell) with respective threshold has been predicted to indicate such expressed RNAs turns out to be lncRNAs, corresponding with the general lower lncRNA expression pattern comparing to mRNAs [62]. As for the predicted exclusive expression pattern of lncRNAs, although various publications inferred that the expression specificity of lncRNAs are lower than that of mRNAs [63,64,65], an independent study [66] reported in 2016, implied that lncRNAs should have higher expression specificity than mRNAs and the identified lack of tissue specificity and exclusivity of lncRNAs can be attribute to the high detection limit of RNA sequencing/detection approaches and the low expression pattern of such cluster of RNAs. As for the second rule (Rule-2), similarly with the first rule, the expression level of our target RNA, expression level lower than respective threshold in neuronal stem cell (2.58), hepatocyte (0) and mast cell (0.151) simultaneously turns out to be lncRNA. According to recent publications, in 2013, a systematic research on the expression level of lncRNAs in multiple neural stem cells confirmed that the average general expression of lncRNAs in such cell subtypes are lower than our parameter, thereby validating this rule [67].

The third and fourth, fifth rules (Rule-3, Rule-4 and Rule-5) all involve neuronal stem cell, hepatocyte, intestinal epithelial cell, mesenchymal cell. According to such rules, quite lower expression level of our target RNAs in such cells may indicate such RNAs turn out to be lncRNAs, corresponding with general expression tendency. Take a specific parameter of intestinal epithelial cell expression pattern of rule three as an example. Two independent studies [68,69] on the expression pattern of lncRNAs in intestinal epithelial cells confirmed that generally, the expression level of such RNAs are lower than mRNAs and RNAs expression level lower than 0.5 tend to be lncRNAs, corresponding with these rules. Further, RNAs with expression level lower than 2 in mesenchymal cells have also been indirectly validated by a specific study on mRNA expression analysis of mesenchymal cells [70]. Therefore, as we have discussed above, the first five rules for distinguishing lncRNAs and mRNAs can be validated by recent publications, reflecting the reasonability of screened rules and indicating that lncRNAs and mRNAs definitely have distinctive expression level inclination.

## 4. Materials and Methods

### 4.1. Dataset and Feature Representation

To construct a reliable dataset, we selected 9339 lncRNAs and 14,294 mRNAs from Hon et al.’s study [24], wherein all RNAs were validated and evaluated by experimental bioinformatics methods. Each RNA sample in the dataset was encoded by 71 expression features, including 69 maximum expression features for 69 types of cells, one feature for the maximum expression in all cells, and one expression specificity feature [24]. Based on Hon et al. [24], the expression values of Counts Per Million (CPM) were from FANTOM5 cap analysis of gene expression (CAGE) data. The expression levels were normalized across samples with edgeR. The 69 maximum expression features for 69 types of cells and the one feature for the maximum expression in all cells were the corresponding CPM expression levels. The expression specificity feature was measured as Chao-Shen-corrected Shannon’s entropy of the CPM expression levels. Based on the encoded RNA samples, we can explore the different expression patterns of lncRNAs and mRNAs in various cell types. To perform this method, we built a binary classification problem, treating lncRNAs as positive samples and mRNAs as negative samples.

### 4.2. Feature Selection Method

Each lncRNA or mRNA in our dataset was encoded by 71 expression features as described in Section 4.1. Clearly, not all features contributed equally to discriminating these two types of RNAs, i.e., some of them may play more important roles than others. Thus, we performed a two-stage feature selection method to investigate the association between features and RNA types.

#### 4.2.1. mRMR Method

In the first stage, all features were ranked by a powerful feature selection method, i.e., the mRMR method [25]. This method has been widely used to rank features in several studies [71,72,73,74,75,76,77,78,79,80]. In this method, two criteria, namely, (1) Max-Relevance and (2) Min-Redundancy, were adopted to rank features singly or simultaneously. If only the first one was used, features are sorted according to their relevance to target variable, resulting in a feature list, named MaxRel feature list. Meanwhile, if both of two criteria were used, i.e., each feature is evaluated according to the relevance between it and target variable and also the redundancies to other features, another feature list, called mRMR feature list, can be produced. Therefore, users can obtain two output feature lists: (1) MaxRel feature list and (2) mRMR feature list, via the mRMR method. In this study, we not only extracted important features that can mark the differences between lncRNAs and mRNAs but also built a good classifier for discriminating these two types of RNAs. Thus, we only used the second list, i.e., the mRMR feature list. In this list, features with high ranks must have high relevance to target variable and low redundancies to features that are listed before it.

After the mRMR method had been applied to analyze the data of lncRNAs and mRNAs, we obtained the mRMR feature list, denoted as the following:

(1) $$F=\left[F_{1},F_{2},\ldots ,\;F_{N}\right]\;$$

where *N* is the total number of features (*N* = 71 in this study). A detailed description on the mRMR method and how to construct the mRMR feature list can be found in Peng et al.’s study [25]. In this study, we used the mRMR program downloaded at http://home.penglab.com/proj/mRMR/index.htm and its default parameters were used.

#### 4.2.2. IFS Method

In the second stage, we further used the mRMR feature list derived from the mRMR method. However, the mRMR feature list only sorts features according to their importance, which features are more important than others is still a problem. Here, the IFS method [26] was adopted to determine which features were important. This method first produced several feature subsets, denoted as:

(2) $$\;FS_{i}=\left\{F_{1},F_{2},\ldots ,F_{i}\right\}\;\left(1\leq i\leq N\right),\;$$

Notably, $FS_{i}$ consists the first *i* features in *F*. Then, for each *FS_i_*, all RNAs mentioned in Section 4.1 were encoded by features in *FS_i_*, and a classification algorithm was performed on this dataset, evaluated by a 10-fold cross-validation [81,82,83,84,85]. After all feature subsets had been tested, the feature subset that can yield the best performance was selected. Optimal features were defined as features in this set, and the classifier with these optimal features was termed as optimal classifier.

### 4.3. Classification Algorithm

In this study, we employed two classification algorithms: RF [27] and RIPPER [28], where RF was adopted to build a classifier with good performance, while RIPPER was used to extract informative rules that can clearly indicate the difference between lncRNAs and mRNAs. Their brief descriptions were as follows.

RF is an integrated classifier. RF always comprises several decision trees [27]. In the training stage, two statistical techniques, namely, bootstrap method [86], and random selection of features [87] were adopted to create decision trees for a training dataset with *N* samples. In the bootstrap procedure, *N* samples were randomly selected from the training dataset, with replacement, to constitute a new dataset. This way, *B* (*B* is a parameter indicating the number of decision trees) datasets can be constructed. For each constructed dataset with *N* samples, a decision tree can be built. Random selection of features is adopted in the construction procedure of decision trees. We randomly selected some features, which are much lesser than total features, and determined the optimal splitting way to extend a tree at one node. Finally, *B* decision trees can be built. For a query sample, each decision tree provides a predicted result. RF integrates these results by using majority voting. RF is deemed to be an effective classification algorithm and has been adopted to tackle different biological problems to date [85,88,89,90,91,92].

**Figure 4:**
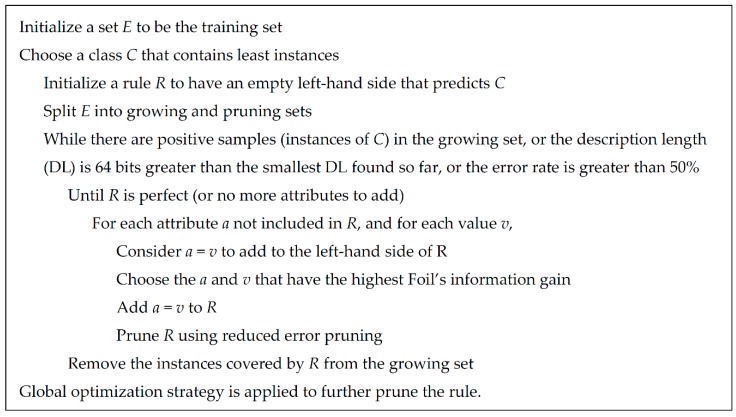
The rule learning procedures of repeated incremental pruning to produce error reduction (RIPPER) algorithm [85].

RIPPER [28], proposed by Cohen in 1995, is a classic rule learning algorithm based on rough set theory. It is an optimized version of Incremental Reduced Error Pruning (IREP) [93]. Compared with C4.5, RIPPER is much more efficient on large datasets and provides competitive results. Figure 4 illustrates the detailed procedures of rule learning using RIPPER. The output rules of RIPPER always contain the conditions, listed in the left hand, and the outcome, listed in the right hand. For example, (Neuronal stem cell ≤ 2.58) and (Hepatocyte ≤ 0) and (Mast cell ≤ 0.151) ≥ class = lncRNA. 

Weka [94] (version 3.8.0) is a powerful suit of software, collecting several state-of-the-art machine learning algorithms and tools, which can be downloaded at https://www.cs.waikato.ac.nz/ml/weka/. One classifier in Weka, called “RandomForest”, implements the abovementioned RF algorithm. And the other classifier “JRip” implements the RIPPER algorithm. Here, these two tools were directly employed in this study and were used with their default setting.

### 4.4. Performance Measurements

Several classifiers with different classification algorithms were constructed on features in different feature subsets. The 10-fold cross-validation [95] was adopted to evaluate the prediction performance of each classifier. As a binary classification problem, four values can be counted according to the predicted results: (1) True positive (*TP*); (2) True negative (*TN*); (3) False positive (*FP*); and (4) False negative (*FN*).

Based on the abovementioned values, four measurements, named *SN*, *SP*, *ACC* [96,97], and *MCC* [98], can be computed to evaluate the prediction ability of each classifier. Their definitions are as follows:

(3) $$SN=\frac{TP}{TP+FN}\;$$

(4) $$SP=\frac{TN}{TN+FP}\;$$

(5) $$ACC=\frac{TP+TN}{TP+TN+FP+FN}\;$$

(6) $$MCC=\frac{TP\times TN-FP\times FN}{\sqrt{\left(TP+FP)(TP+FN)(TN+FP)(TN+FN\right)}}\;$$

Among the four measurements, *MCC* is a comprehensive measurement ranging from −1 to 1. *MCC* can more accurately evaluate the performance of each classifier than *ACC* because *MCC* is a balanced measure even if the sizes of classes are very different. Thus, *MCC* was selected as a major one in this study, whereas other measurements were provided as references.

## 5. Conclusions

Based on the expression data of lncRNA and mRNA retrieved from a recent publication, we proposed a computational scheme to identify a group of functional features and rules, corresponding to cell subtypes, which have distinctive expression patterns of such two RNA subtypes. According to recent publications, several top features can be confirmed to have such function and obtained rules can really indicate the different expression patterns on two RNA subtypes. The new findings of this study may not only reveal the heterogeneous expression patterns of lncRNA and mRNA but also provide us a new tool to identify the potential biological functions of different RNA types. In addition, we provided the whole dataset investigated in this study in Supplementary Data 1, with which readers can easily replicate our work.

## Figures and Tables

**Table 1:** Performance of the optimal random forest (RF), repeated incremental pruning to produce error reduction (RIPPER), nearest neighbor algorithm (1-NN), support vector machine (SVM), and logistic regression (LR) classifier.

Classification Algorithm	Number of Used Features	SN	SP	ACC	MCC
RF	53	**0.963**	0.940	**0.949**	**0.895**
RIPPER	70	0.952	**0.942**	0.946	0.888
1-NN	69	0.932	0.896	0.911	0.818
SVM	71	0.758	0.861	0.820	0.622
LR	19	0.944	0.876	0.903	0.806

**Table 2:** Top 13 features in maximum relevance minimum redundancy (mRMR) feature list.

No.	Feature Name
1	Expression specificity
2	Intestinal epithelial cell
3	Neutrophil
4	Hepatocyte
5	Mast cell
6	Fibroblast of the conjuctiva
7	Reticulocyte
8	Mesenchymal cell
9	Lymphocyte of b lineage
10	Neuronal stem cell
11	Macrophage
12	Pericyte cell
13	Max cpm in all facet

**Table 3:** 18 Classification rules yielded by repeated incremental pruning to produce error reduction (RIPPER) on top ten features.

Rule Number	Condition	Outcome
Rule-1	(Neuronal stem cell ≤ 2.58) and (Mesenchymal cell ≤ 1.44) and (Intestinal epithelial cell ≤ 0) and (Mesenchymal cell ≤ 0.197) and (Expression specificity ≥ 0.552495)	lncRNA
Rule-2	(Neuronal stem cell ≤ 2.58) and (Hepatocyte ≤ 0) and (Mast cell ≤ 0.151)	lncRNA
Rule-3	(Neuronal stem cell ≤ 2.58) and (Hepatocyte ≤ 0) and (Intestinal epithelial cell ≤ 0.246) and (Mesenchymal cell ≤ 2.04) and (Neuronal stem cell ≤ 0)	lncRNA
Rule-4	(Neuronal stem cell ≤ 5.17) and (Mast cell ≤ 0.842) and (Intestinal epithelial cell ≤ 0.737) and (Neuronal stem cell ≤ 0.542) and (Mesenchymal cell ≤ 2.75)	lncRNA
Rule-5	(Neuronal stem cell ≤ 2.58) and (Hepatocyte ≤ 1.73) and (Intestinal epithelial cell ≤ 0.246) and (Mesenchymal cell ≤ 2.63) and (Neuronal stem cell ≤ 0)	lncRNA
Rule-6	(Neuronal stem cell ≤ 2.58) and (Hepatocyte ≤ 1.73) and (Mast cell ≤ 0.352) and (Expression specificity ≤ 0.299388) and (Mast cell ≤ 0)	lncRNA
Rule-7	(Neuronal stem cell ≤ 5.17) and (Hepatocyte ≤ 1.73) and (Intestinal epithelial cell ≤ 0.246) and (Expression specificity ≥ 0.497214) and (Lymphocyte of b lineage ≥ 1.23)	lncRNA
Rule-8	(Neuronal stem cell ≤ 2.58) and (Hepatocyte ≤ 1.73) and (Mesenchymal cell ≤ 1.44) and (Intestinal epithelial cell ≤ 0)	lncRNA
Rule-9	(Neuronal stem cell ≤ 5.17) and (Mesenchymal cell ≤ 4.52) and (Reticulocyte ≤ 0) and (Fibroblast of the conjuctiva ≤ 0) and (Hepatocyte ≤ 3.46) and (Expression specificity ≥ 0.311106) and (Intestinal epithelial cell ≤ 4.18) and (Neuronal stem cell ≤ 2.71)	lncRNA
Rule-10	(Neuronal stem cell ≤ 5.17) and (Mast cell ≤ 0.842) and (Intestinal epithelial cell ≤ 1.72) and (Hepatocyte ≤ 0) and (Fibroblast of the conjuctiva ≤ 0)	lncRNA
Rule-11	(Neuronal stem cell ≤ 5.17) and (Hepatocyte ≤ 5.19) and (Intestinal epithelial cell ≤ 1.23) and (Neuronal stem cell ≤ 0.542) and (Mesenchymal cell ≤ 10.1) and (Mast cell ≤ 0.907)	lncRNA
Rule-12	(Neuronal stem cell ≤ 2.58) and (Mesenchymal cell ≤ 4.5) and (Intestinal epithelial cell ≤ 0.983) and (Hepatocyte ≤ 0) and (Fibroblast of the conjuctiva ≤ 0)	lncRNA
Rule-13	(Neuronal stem cell ≤ 5.17) and (Mesenchymal cell ≤ 5.18) and (Mesenchymal cell ≤ 1.45) and (Neutrophil ≥ 0.848) and (Expression specificity ≤ 0.333673) and (Mesenchymal cell ≤ 0.904) and (Fibroblast of the conjuctiva ≤ 0)	lncRNA
Rule-14	(Neuronal stem cell ≤ 5.17) and (Mesenchymal cell ≤ 5.18) and (Neuronal stem cell ≤ 2.58) and (Intestinal epithelial cell ≤ 0.737) and (Neuronal stem cell ≤ 0) and (Expression specificity ≤ 0.517863) and (Mesenchymal cell ≥ 2.87)	lncRNA
Rule-15	(Neuronal stem cell ≤ 5.17) and (Mast cell ≤ 1.06) and (Hepatocyte ≤ 5.19) and (Neuronal stem cell ≤ 0.542) and (Intestinal epithelial cell ≤ 6.88) and (Mesenchymal cell ≤ 28.5)	lncRNA
Rule-16	(Neuronal stem cell ≤ 5.17) and (Mesenchymal cell ≤ 5.28) and (Fibroblast of the conjuctiva ≤ 0) and (Mast cell ≤ 0.842) and (Intestinal epithelial cell ≤ 11.8) and (Neuronal stem cell ≤ 2.58) and (Hepatocyte ≤ 20.1)	lncRNA
Rule-17	(Neuronal stem cell ≤ 7.75) and (Intestinal epithelial cell ≤ 1.72) and (Mesenchymal cell ≤ 4.52) and (Neuronal stem cell ≤ 2.58) and (Lymphocyte of b lineage ≤ 4.07) and (Neutrophil ≥ 5.72) and (Mast cell ≤ 19) and (Expression specificity ≥ 0.115115)	lncRNA
Rule-18	Otherwise	mRNA

## Supplementary Materials

The following are available online at http://www.mdpi.com/1422-0067/19/11/3416/s1.
