# Supplementary material for: Tissue Expression Difference between mRNAs and lncRNAs
Source: Int J Mol Sci. 2018 Oct 31;19(11):3416. doi: 10.3390/ijms19113416 (PMC6274976; doi:10.3390/ijms19113416)
Supplement: Supplementary file 1 [file ijms-19-03416-s001.zip › Table-S1.pdf]

Table S1. mRMR feature list yielded by the mRMR method.

Rank	Feature name
1	Expression specificity
2	Intestinal epithelial cell
3	Neutrophil
4	Hepatocyte
5	Mast cell
6	Fibroblast of the conjuctiva
7	Reticulocyte
8	Mesenchymal cell
9	Lymphocyte of b lineage
10	Neuronal stem cell
11	Macrophage
12	Pericyte cell
13	Max cpm in all facet
14	Endothelial cell of lymphatic vessel
15	Natural killer cell
16	Chondrocyte
17	Placental epithelial cell
18	Cardiac myocyte
19	T cell
20	Epithelial cell of prostate

21	Monocyte
22	Mesothelial cell
23	Neuron
24	Dendritic cell
25	Corneal epithelial cell
26	Fibroblast of lymphatic vessel
27	Basophil
28	Fat cell
29	Melanocyte
30	Tendon cell
31	Bronchial smooth muscle cell
32	Epithelial cell of malassez
33	Fibroblast of choroid plexus
34	Acinar cell
35	Trabecular meshwork cell
36	Endothelial cell of hepatic sinusoid
37	Mammary epithelial cell
38	Sensory epithelial cell
39	Smooth muscle cell of the esophagus
40	Gingival epithelial cell
41	Hepatic stellate cell
42	Preadipocyte

43	Keratocyte
44	Retinal pigment epithelial cell
45	Hair follicle cell
46	Lens epithelial cell
47	Urothelial cell
48	Amniotic epithelial cell
49	Blood vessel endothelial cell
50	Vascular associated smooth muscle cell
51	Fibroblast of gingiva
52	Myoblast
53	Respiratory epithelial cell
54	Keratinocyte
55	Uterine smooth muscle cell
56	Kidney epithelial cell
57	Smooth muscle cell of trachea
58	Astrocyte
59	Epithelial cell of esophagus
60	Fibroblast of pulmonary artery
61	Cardiac fibroblast
62	Ciliated epithelial cell
63	Skin fibroblast
64	Osteoblast

65	Smooth muscle cell of prostate
66	Fibroblast of periodontium
67	Skeletal muscle cell
68	Iris pigment epithelial cell
69	Stromal cell
70	Enteric smooth muscle cell
71	Fibroblast of tunica adventitia of artery
