# Supplementary material for: Tissue Expression Difference between mRNAs and lncRNAs
Source: Int J Mol Sci. 2018 Oct 31;19(11):3416. doi: 10.3390/ijms19113416 (PMC6274976; doi:10.3390/ijms19113416)
Supplement: Supplementary file 1 [file ijms-19-03416-s001.zip › Table-S2.pdf]

Table S2. SNs, SPs, ACCs, and MCCs obtained by the IFS method and RF algorithm.

Number of features	SN	SP	ACC	MCC
4	0.857	0.899	0.883	0.755
5	0.886	0.909	0.900	0.792
6	0.898	0.915	0.908	0.809
7	0.901	0.916	0.910	0.813
8	0.919	0.918	0.919	0.831
9	0.922	0.917	0.919	0.833
10	0.947	0.932	0.938	0.872
11	0.945	0.932	0.937	0.869
12	0.948	0.933	0.939	0.874
13	0.953	0.936	0.943	0.882
14	0.953	0.935	0.942	0.881
15	0.953	0.937	0.943	0.883
16	0.954	0.937	0.944	0.885
17	0.953	0.936	0.943	0.883
18	0.954	0.936	0.943	0.883
19	0.954	0.937	0.944	0.884
20	0.956	0.935	0.943	0.883
21	0.956	0.937	0.944	0.886
22	0.957	0.938	0.946	0.889

23	0.957	0.939	0.946	0.888
24	0.958	0.938	0.946	0.889
25	0.959	0.938	0.946	0.890
26	0.959	0.938	0.946	0.890
27	0.958	0.939	0.946	0.890
28	0.957	0.938	0.946	0.888
29	0.959	0.938	0.946	0.890
30	0.959	0.939	0.947	0.891
31	0.960	0.937	0.946	0.890
32	0.961	0.938	0.947	0.892
33	0.958	0.937	0.946	0.888
34	0.959	0.938	0.946	0.889
35	0.960	0.938	0.946	0.890
36	0.960	0.938	0.947	0.891
37	0.959	0.937	0.946	0.889
38	0.959	0.937	0.946	0.888
39	0.959	0.938	0.946	0.890
40	0.959	0.938	0.946	0.889
41	0.959	0.937	0.946	0.888
42	0.959	0.939	0.947	0.890
43	0.959	0.938	0.946	0.890
44	0.960	0.938	0.947	0.891

45	0.962	0.938	0.948	0.893
46	0.961	0.941	0.949	0.894
47	0.962	0.939	0.948	0.893
48	0.960	0.940	0.948	0.893
49	0.960	0.940	0.948	0.892
50	0.959	0.939	0.947	0.891
51	0.962	0.939	0.948	0.893
52	0.963	0.939	0.949	0.894
53	0.963	0.940	0.949	0.895
54	0.963	0.939	0.949	0.894
55	0.961	0.938	0.947	0.892
56	0.960	0.939	0.947	0.892
57	0.961	0.938	0.947	0.891
58	0.961	0.939	0.947	0.892
59	0.962	0.939	0.948	0.894
60	0.959	0.940	0.947	0.892
61	0.962	0.940	0.949	0.895
62	0.961	0.938	0.947	0.891
63	0.963	0.938	0.948	0.893
64	0.960	0.939	0.947	0.892
65	0.961	0.938	0.947	0.891
66	0.958	0.937	0.946	0.888

67	0.961	0.938	0.947	0.890
68	0.959	0.938	0.947	0.890
69	0.959	0.940	0.947	0.892
70	0.960	0.940	0.948	0.892
71	0.961	0.937	0.946	0.890
