# Supplementary material for: Tissue Expression Difference between mRNAs and lncRNAs
Source: Int J Mol Sci. 2018 Oct 31;19(11):3416. doi: 10.3390/ijms19113416 (PMC6274976; doi:10.3390/ijms19113416)
Supplement: Supplementary file 1 [file ijms-19-03416-s001.zip › Table-S3.pdf]

Table S3. SNs, SPs, ACCs, and MCCs obtained by the IFS method and RIPPER algorithm.

**Number of features**	**SN**	**SP**	**ACC**	**MCC**
4	0.925	0.903	0.912	0.819
5	0.934	0.907	0.917	0.831
6	0.936	0.914	0.923	0.842
7	0.934	0.916	0.923	0.842
8	0.934	0.920	0.926	0.847
9	0.932	0.920	0.925	0.844
10	0.945	0.937	0.940	0.876
11	0.945	0.935	0.939	0.874
12	0.948	0.938	0.942	0.880
13	0.946	0.942	0.943	0.883
14	0.949	0.938	0.942	0.880
15	0.946	0.941	0.943	0.882
16	0.946	0.938	0.941	0.879
17	0.947	0.941	0.943	0.883
18	0.946	0.940	0.942	0.881
19	0.949	0.938	0.943	0.881
20	0.948	0.939	0.942	0.881
21	0.946	0.941	0.943	0.882
22	0.949	0.941	0.944	0.884
23	0.949	0.939	0.943	0.883
24	0.951	0.942	0.945	0.887
25	0.950	0.939	0.943	0.882
26	0.950	0.941	0.945	0.885
27	0.949	0.942	0.945	0.886
28	0.950	0.939	0.944	0.883
29	0.949	0.940	0.944	0.884
30	0.949	0.939	0.943	0.882
31	0.947	0.941	0.943	0.883
32	0.951	0.939	0.944	0.883
33	0.948	0.941	0.943	0.883
34	0.950	0.939	0.943	0.883
35	0.952	0.940	0.944	0.885
36	0.948	0.940	0.943	0.883
37	0.951	0.939	0.943	0.883
38	0.947	0.941	0.943	0.882
39	0.949	0.942	0.944	0.885
40	0.951	0.942	0.946	0.888

41	0.953	0.937	0.944	0.884
42	0.947	0.942	0.944	0.884
43	0.943	0.941	0.942	0.880
44	0.950	0.942	0.945	0.886
45	0.951	0.940	0.944	0.885
46	0.950	0.942	0.945	0.886
47	0.952	0.938	0.944	0.884
48	0.951	0.942	0.946	0.888
49	0.950	0.939	0.943	0.883
50	0.950	0.942	0.945	0.886
51	0.954	0.940	0.946	0.888
52	0.953	0.940	0.945	0.887
53	0.953	0.939	0.944	0.885
54	0.949	0.941	0.944	0.884
55	0.948	0.941	0.944	0.883
56	0.950	0.941	0.944	0.885
57	0.950	0.940	0.944	0.884
58	0.949	0.941	0.944	0.884
59	0.949	0.942	0.945	0.886
60	0.951	0.941	0.945	0.886
61	0.948	0.941	0.944	0.884
62	0.949	0.942	0.944	0.885
63	0.946	0.941	0.943	0.881
64	0.948	0.942	0.944	0.885
65	0.951	0.942	0.946	0.887
66	0.951	0.941	0.945	0.887
67	0.951	0.941	0.945	0.887
68	0.954	0.940	0.945	0.887
69	0.949	0.940	0.944	0.883
70	0.952	0.942	0.946	0.888
71	0.948	0.942	0.944	0.885
