# Supplementary material for: Tissue Expression Difference between mRNAs and lncRNAs
Source: Int J Mol Sci. 2018 Oct 31;19(11):3416. doi: 10.3390/ijms19113416 (PMC6274976; doi:10.3390/ijms19113416)
Supplement: Supplementary file 1 [file ijms-19-03416-s001.zip › Table-S4.pdf]

Table S4. SNs, SPs, ACCs, and MCCs obtained by the IFS method and 1-NN, SVM or LR algorithm.

(1) SNs, SPs, ACCs, and MCCs obtained by the IFS method and 1-NN algorithm.

Number of features	SN	SP	ACC	MCC
4	0.850	0.860	0.856	0.703
5	0.859	0.865	0.863	0.717
6	0.874	0.869	0.871	0.734
7	0.876	0.868	0.871	0.736
8	0.885	0.869	0.875	0.744
9	0.885	0.869	0.875	0.745
10	0.918	0.894	0.903	0.803
11	0.918	0.894	0.903	0.802
12	0.919	0.895	0.904	0.804
13	0.921	0.898	0.907	0.810
14	0.921	0.897	0.906	0.808
15	0.923	0.896	0.907	0.809
16	0.923	0.896	0.907	0.809
17	0.922	0.895	0.906	0.807
18	0.923	0.895	0.906	0.807
19	0.922	0.895	0.905	0.807
20	0.922	0.895	0.906	0.807
21	0.922	0.897	0.907	0.809

22	0.924	0.896	0.907	0.810
23	0.926	0.898	0.909	0.815
24	0.927	0.898	0.909	0.815
25	0.928	0.898	0.910	0.816
26	0.928	0.898	0.910	0.815
27	0.927	0.897	0.909	0.815
28	0.928	0.897	0.909	0.815
29	0.929	0.897	0.910	0.816
30	0.928	0.897	0.910	0.816
31	0.929	0.897	0.910	0.816
32	0.929	0.897	0.910	0.816
33	0.929	0.897	0.910	0.816
34	0.929	0.897	0.909	0.815
35	0.929	0.897	0.909	0.815
36	0.929	0.897	0.910	0.816
37	0.928	0.897	0.909	0.815
38	0.928	0.897	0.909	0.815
39	0.928	0.897	0.909	0.815
40	0.928	0.897	0.909	0.815
41	0.928	0.897	0.910	0.816
42	0.929	0.897	0.910	0.816
43	0.928	0.897	0.909	0.815

44	0.930	0.896	0.910	0.816
45	0.929	0.896	0.909	0.815
46	0.930	0.896	0.910	0.816
47	0.931	0.896	0.910	0.816
48	0.931	0.896	0.910	0.816
49	0.931	0.895	0.909	0.815
50	0.931	0.895	0.909	0.815
51	0.931	0.895	0.910	0.816
52	0.932	0.895	0.910	0.816
53	0.931	0.895	0.909	0.816
54	0.931	0.895	0.910	0.816
55	0.930	0.895	0.909	0.815
56	0.931	0.896	0.910	0.816
57	0.932	0.896	0.910	0.817
58	0.932	0.896	0.910	0.817
59	0.931	0.896	0.910	0.817
60	0.932	0.896	0.910	0.817
61	0.932	0.896	0.910	0.817
62	0.932	0.895	0.910	0.816
63	0.932	0.895	0.910	0.817
64	0.932	0.896	0.910	0.817
65	0.932	0.896	0.910	0.817

66	0.931	0.896	0.910	0.817
67	0.932	0.896	0.910	0.817
68	0.932	0.896	0.910	0.818
69	0.932	0.896	0.911	0.818
70	0.931	0.896	0.910	0.817
71	0.933	0.896	0.910	0.818

(2) SNs, SPs, ACCs, and MCCs obtained by the IFS method and SVM algorithm.

Number of features	SN	SP	ACC	MCC
4	0.705	0.859	0.798	0.574
5	0.709	0.860	0.800	0.577
6	0.711	0.858	0.800	0.578
7	0.712	0.858	0.801	0.579
8	0.712	0.858	0.801	0.579
9	0.714	0.858	0.801	0.580
10	0.717	0.860	0.803	0.584
11	0.718	0.860	0.804	0.586
12	0.719	0.860	0.804	0.586
13	0.722	0.862	0.807	0.592
14	0.725	0.862	0.808	0.595
15	0.727	0.862	0.809	0.596
16	0.728	0.862	0.809	0.597
17	0.729	0.862	0.809	0.598

18	0.729	0.862	0.809	0.598
19	0.730	0.862	0.810	0.599
20	0.734	0.862	0.812	0.603
21	0.735	0.862	0.812	0.604
22	0.737	0.862	0.813	0.606
23	0.739	0.862	0.813	0.607
24	0.739	0.863	0.814	0.608
25	0.740	0.863	0.814	0.608
26	0.740	0.863	0.814	0.608
27	0.741	0.863	0.814	0.609
28	0.741	0.862	0.814	0.609
29	0.741	0.862	0.814	0.609
30	0.741	0.862	0.814	0.609
31	0.742	0.862	0.815	0.609
32	0.742	0.862	0.815	0.610
33	0.742	0.862	0.815	0.610
34	0.742	0.862	0.815	0.610
35	0.742	0.862	0.815	0.610
36	0.743	0.862	0.815	0.610
37	0.743	0.863	0.815	0.611
38	0.744	0.863	0.816	0.612
39	0.744	0.863	0.816	0.612

40	0.744	0.863	0.816	0.612
41	0.744	0.863	0.816	0.612
42	0.744	0.863	0.816	0.612
43	0.744	0.863	0.816	0.612
44	0.744	0.863	0.816	0.613
45	0.745	0.863	0.816	0.613
46	0.745	0.863	0.816	0.613
47	0.746	0.863	0.817	0.614
48	0.746	0.863	0.817	0.614
49	0.746	0.863	0.817	0.614
50	0.747	0.863	0.817	0.615
51	0.748	0.862	0.817	0.615
52	0.748	0.862	0.817	0.615
53	0.748	0.862	0.817	0.615
54	0.749	0.862	0.818	0.616
55	0.749	0.862	0.818	0.616
56	0.750	0.862	0.818	0.616
57	0.750	0.862	0.818	0.617
58	0.751	0.862	0.818	0.618
59	0.751	0.862	0.818	0.618
60	0.751	0.862	0.818	0.618
61	0.751	0.862	0.818	0.618

62	0.752	0.862	0.819	0.619
63	0.753	0.862	0.819	0.619
64	0.754	0.862	0.819	0.619
65	0.754	0.862	0.819	0.620
66	0.755	0.861	0.819	0.620
67	0.755	0.862	0.820	0.621
68	0.755	0.861	0.820	0.621
69	0.756	0.861	0.820	0.621
70	0.757	0.861	0.820	0.622
71	0.758	0.861	0.820	0.622

(3) SNs, SPs, ACCs, and MCCs obtained by the IFS method and LR algorithm.

Number of features	SN	SP	ACC	MCC
4	0.898	0.844	0.865	0.729
5	0.896	0.849	0.868	0.733
6	0.951	0.812	0.867	0.746
7	0.954	0.814	0.869	0.751
8	0.906	0.839	0.865	0.731
9	0.796	0.873	0.843	0.670
10	0.947	0.840	0.883	0.771
11	0.949	0.846	0.887	0.779
12	0.922	0.876	0.894	0.786
13	0.929	0.885	0.902	0.802

14	0.889	0.897	0.894	0.780
15	0.881	0.897	0.890	0.773
16	0.903	0.889	0.894	0.783
17	0.898	0.891	0.893	0.781
18	0.933	0.875	0.898	0.795
19	0.944	0.876	0.903	0.806
20	0.915	0.881	0.894	0.785
21	0.830	0.906	0.876	0.740
22	0.789	0.907	0.860	0.706
23	0.758	0.897	0.842	0.666
24	0.761	0.892	0.840	0.662
25	0.799	0.879	0.848	0.680
26	0.804	0.878	0.849	0.683
27	0.783	0.880	0.842	0.667
28	0.774	0.881	0.839	0.661
29	0.793	0.879	0.845	0.675
30	0.825	0.870	0.852	0.692
31	0.844	0.868	0.858	0.706
32	0.921	0.866	0.888	0.774
33	0.932	0.882	0.902	0.802
34	0.936	0.878	0.901	0.802
35	0.940	0.876	0.901	0.802

36	0.936	0.869	0.895	0.791
37	0.908	0.879	0.891	0.777
38	0.920	0.878	0.894	0.786
39	0.884	0.867	0.874	0.742
40	0.864	0.878	0.873	0.737
41	0.890	0.877	0.882	0.758
42	0.842	0.883	0.867	0.723
43	0.862	0.885	0.876	0.742
44	0.745	0.898	0.837	0.656
45	0.749	0.895	0.837	0.656
46	0.759	0.889	0.837	0.656
47	0.807	0.881	0.852	0.689
48	0.708	0.887	0.816	0.611
49	0.762	0.858	0.820	0.623
50	0.784	0.842	0.819	0.624
51	0.806	0.849	0.832	0.651
52	0.868	0.816	0.836	0.671
53	0.899	0.804	0.841	0.688
54	0.897	0.824	0.853	0.707
55	0.940	0.773	0.839	0.697
56	0.942	0.757	0.830	0.685
57	0.922	0.792	0.844	0.699

58	0.910	0.882	0.893	0.781
59	0.933	0.877	0.899	0.798
60	0.931	0.880	0.900	0.799
61	0.930	0.882	0.901	0.800
62	0.916	0.883	0.896	0.788
63	0.880	0.890	0.886	0.764
64	0.875	0.892	0.886	0.763
65	0.925	0.883	0.900	0.797
66	0.905	0.887	0.894	0.783
67	0.852	0.897	0.879	0.748
68	0.888	0.887	0.887	0.767
69	0.861	0.894	0.881	0.751
70	0.800	0.904	0.863	0.712
71	0.777	0.908	0.856	0.696
